# Lead-free, formamidinium germanium-antimony halide (FA_4_GeSbCl_12_) double perovskite solar cells: the effects of band offsets†

**DOI:** 10.1039/d3ra03102k

**Published:** 2023-08-25

**Authors:** Karthick Sekar, Latha Marasamy, Sasikumar Mayarambakam, Hesham Hawashin, Mohamad Nour, Johann Bouclé

**Affiliations:** a Univ. Limoges, CNRS, XLIM, UMR 7252 Limoges F-87000 France s.karthick.dsc@gmail.com; b GREMAN UMR 7347, Université de Tours, CNRS, INSA Centre Val de Loire 37071 Tours France; c Facultad de Química, Materiales-Energía, Universidad Autónoma de Querétaro Santiago de Querétaro Querétaro C.P. 76010 Mexico; d Department of Chemistry, Indian Institute of Science Education and Research (IISER)-Tirupati Tirupati 517507 A.P. India sasikumarm@iisertirupati.ac.in

## Abstract

Double halide perovskites have received massive attention due to their low toxicity, tunable bandgap, structural flexibility, and stability as compared to conventional 3D lead halide perovskites. Particularly, newly discovered formamidinium germanium-antimony halide (FA_4_GeSbCl_12_) double perovskites offer an excellent bandgap (∼1.3 eV) for solar cell (SC) applications. Therefore, in this study, for the first time, we have simulated FTO/TiO_2_/FA_4_GeSbCl_12_/Cu_2_O/Au planar SCs using SCAPS-1D, showing a maximum power conversion efficiency of 22.5% with *J*_sc_ = 34.52 mA cm^−2^, *V*_oc_ = 0.76 V, and FF = 85.1%. The results show that the variation in valence and conduction band offsets (−0.4 to +0.2 eV and −0.4 to +0.57 eV) at the ETL/absorber and absorber/HTL interfaces dominate the SC performance. Also, different absorber defect densities (1 × 10^14^–1 × 10^20^ cm^−3^) and thicknesses (200–3000 nm) effectively influence the PCE. Moreover, simulated impedance spectroscopy (IS) data (through SCAPS-1D) were fitted using equivalent electrical circuits to extract relevant parameters, including *R*_s_, *R*_HF_, and *R*_LF_, allowing us to better discuss the physics of the device. The fitted IS results strongly revealed that enhanced SC performance is associated with higher recombination resistance and a larger recombination lifetime. Likewise, a slight variation in the *R*_s_ (0 to 2.5 Ω cm^2^) highly impacts the PCE (22.5% to 19.7%). Furthermore, a tandem cell is designed by combining the top cell of ethylenediammonium-FASnI_3_ perovskite with the FA_4_GeSbCl_12_ bottom cell using a filtered spectrum strategy, which opens the door for multi-junction SC applications. These findings firmly reveal that the appropriate energy level alignment at interfaces with suitable material properties is the key to boosting SC performance.

## Abstract 2

We report the numerical simulation of an efficient FA_4_GeSbCl_12_ solar cell with a PCE of 22.5%. This study provides novel device design and explanations to understand device physics mainly based on CBO & VBO at ETL/absorber & absorber/HTL interfaces.

## Graphical abstract

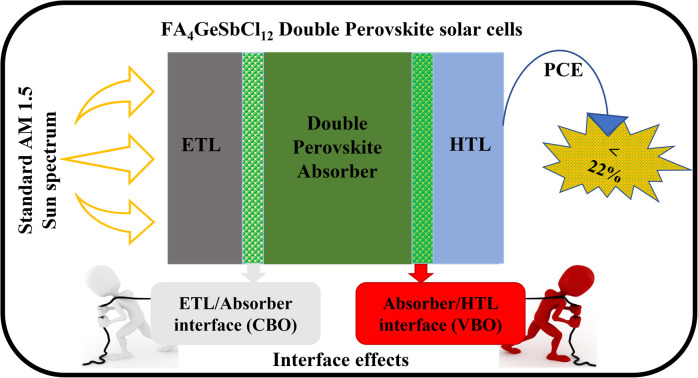

## 1. Introduction

Progress in photovoltaic (PV) technology is increasing substantially, specifically in organic–inorganic perovskite solar cells (PSCs). For instance, the power conversion efficiency (PCE) of single-junction lead-halide perovskites enormously improved from 3 to 25.7% within a decade. Moreover, perovskite/Si and perovskite/CIGS monolithic tandem cells demonstrated PCEs as high as 31.3% and 24.2%, respectively.^1^ This is mainly because of excellent electronic properties, such as high optical absorption coefficient, direct tunable bandgap, long free carrier diffusion lengths, low exciton binding energy, and low defect density.^2,3^ For PV applications, the general formula for three-dimensional (3D) perovskite composition is ABX_3_, where A is a monovalent organic and/or inorganic cation (MA-methylammonium, FA-formamidinium, Cs-cesium and Rb-rubidium) or a mixture, B is an inorganic divalent cation (mainly Pb-lead), and X is a halide anion (chloride (Cl^−^), bromide (Br^−^), iodide (I^−^)), or a mixture of halides.^2,3^ However, different elemental substitutions on A and B sites directly alter the structural dimensionality, including 3D (the usual one), 2D, 1D, or 0D. Usually, the perovskite light absorber in PSCs is sandwiched between electron and hole transport layers (ETL and HTL), followed by front and back contacts, respectively. Apart from the mesoscopic architectures, based on the ETL and HTL arrangements, the device is categorized as planar (n-i-p) and inverted (p-i-n) structures.^4,5^

The presence of Pb is the primary concern in the above-mentioned solar cells as it is toxic to the environment and other living organisms. It also creates severe damage in the human body, including functional disorders in the digestive, blood, and nervous systems.^6^ Conversely, Pb-based perovskites decompose and release harmful soluble salts after a specific time.^7^ Therefore, it is crucial to find non-toxic elements as alternatives. As such, different elements, such as tin (Sn^2+^), germanium (Ge^2+^), antimony (Sb^2+^), and bismuth (Bi^2+^), have been substituted in the B-site instead of Pb.^8,9^ However, these solar cells demonstrated low PCE as compared to Pb-based perovskites. Besides, retaining the device's stability against heat, oxygen, moisture, and light, along with targeted perovskite composition, is a real challenge.^9^

Recently, Pb-free halide double perovskites (LFHDPs) with the formula of A_2_B’B′′X_6_ were discovered in which two bivalent metallic Pb^2+^ cations are substituted by a monovalent and a trivalent cation, altering the dimensionality in the crystal structure.^10–14^ In LFHDPs, multiple cation substitution is one of the most effective strategies due to its compositional and structural flexibility. Goldschmidt tolerance (GT) and octahedral factors (OF) are crucial in finding a suitable and stable perovskite structure. In 2021, Y. Wu *et al.* reported GT and OF values for several LFHDPs.^15^ Among them, only a few perovskites qualified for solar cell applications. Cesium silver bismuth bromide (Cs_2_AgBiBr_6_) is one of the most commonly used absorbers in LFDHPs,^11,15–17^ which showed a PCE as high as 6.4% using hydrogenating treatment wherein the bandgap was tuned from 2.18 to 1.68 eV for a wide range of photon absorption.^18^ However, Cs_2_AgBiBr_6_ has several disadvantages, such as dominant surface defects, strong electron-phonon coupling, the existence of excitons, and difficulties in the fabrication process (*i.e.*, low solubility and high-temperature phase).^11^ On the other hand, recently developed formamidinium germanium-antimony-based halide (FA_4_GeSbCl_12_) double perovskites have an optimum bandgap value of ∼1.3 eV and demonstrated an initial PCE of 4.7% without additives.^19^ Compared to conventional organic–inorganic lead halide perovskites (*i.e.*, MAPbI_3_), the FA_4_GeSbCl_12_ double perovskite has several advantages as follows.^19^ (1) Almost one order of magnitude higher conductivity than MAPbI_3_. (2) Higher thermal stability; for example, FA_4_GeSbCl_12_ double perovskite is stable up to ∼235 °C (also no sign of decomposition for more than 80 days at 60% relative humidity). (3) Higher photostability; for example, there was no sign of structural changes for up to 15 days on exposure to simulated sunlight (100 mW cm^−2^) or UV (360 nm) irradiation. (4) Comparable electron and hole effective masses (0.38 *m*_e_ and 0.18 *m*_h_). (5) Absorption onset occurs at ∼950 nm, demonstrating an efficient band gap (1.3 eV) than MAPbI_3_ (1.55 eV), FAPbI_3_ (1.48 eV), CsPbI_3_ (1.7 eV) for absorbing more photons. Reasons for incorporating Ge^2+^ and Sb^3+^ instead of Pb^2+^ in FA_4_GeSbCl_12_ are as follows: both are less toxic, have lower ionic radii, retain lower and/or similar electronegativity, and are not easily oxidizable. However, only a few reports deal with these compositions to demonstrate working solar cell devices, and our understanding of their interface properties are lacking. In addition, it is crucial to understand the interface properties for further advancement in the solar cell performance of FA_4_GeSbCl_12_ double perovskites.

Therefore, in this work, we have investigated the performance of lead-free FA_4_GeSbCl_12_ double perovskite solar cells through the proposed device design and band offset analysis using drift-diffusion Solar Cell Capacitance Simulator (SCAPS-1D) modeling.^20–25^ The impact of the interfacial properties of valence band offset (VBO) and conduction band offset (CBO) at the ETL/absorber and absorber/HTL interfaces on the solar cell parameters have been systematically studied. The effects of absorber defect density/trap states, thickness, and parasitic resistances (*i.e.*, series and shunt resistance) on the solar cell performance are also explored. We also discuss simulated impedance spectroscopy data using conventional fitting procedures based on equivalent electrical circuits, which is a powerful tool for discussing device operation. As a result, a PCE of 22.5% was achieved from an optimized simulated single-junction solar cell. Furthermore, a tandem architecture was designed using the above-optimized device as a bottom cell and the ethylenediammonium (en)-incorporated formamidinium tin iodide (simply en-FASnl_3_) perovskite absorber as a top cell. This attempt strongly discloses the possibility of employing lead-free FA_4_GeSbCl_12_ double perovskite solar cells in a tandem device structure. To sum up, we firmly believe that our comprehensive study has revealed the effects of VBO and CBO on the performance of lead-free FA_4_GeSbCl_12_ double perovskite solar cells, which are highly beneficial for the experimental scientist to develop efficient devices.

## 2. Device structure and simulation details

**Fig. 1:**
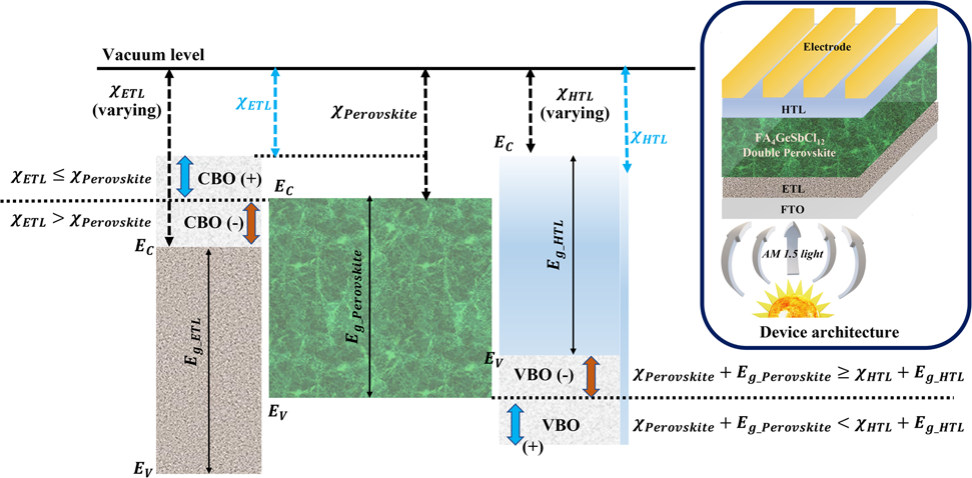
Band alignments at the ETL/absorber and absorber/HTL interfaces. (The inset shows the proposed device structure for this simulation work).

In this study, the drift-diffusion SCAPS-1D simulation software (version 3.3.10) was adopted to investigate the performance of lead-free, formamidinium germanium-antimony halide (FA_4_GeSbCl_12_) double perovskite solar cells. Hereafter, the double perovskite is referred to as DP. As shown in Fig. 1 (inset), the solar cell with the n-i-p configuration consists of fluorine-doped tin oxide (FTO), titanium oxide (TiO_2_), FA_4_GeSbCl_12,_ cuprous oxide (Cu_2_O) and gold (Au), as the transparent conductive oxide, electron transport layer (ETL), absorber layer, hole transport layer (HTL) and metal contact, respectively. Such architecture is chosen for SCAPS-1D modeling since a promising PCE of 4.7% was achieved experimentally in the literature with a device structure based on FTO/TiO_2_/FA_4_GeSbCl_12_/Spiro-OMeTAD/Au.^19^ We used Cu_2_O as an alternative to conventional Spiro-OMeTAD for the following reasons: the energy levels of Spiro-OMeTAD are not well-matched with the DP; Spiro-OMeTAD shows poor stability, relatively poor hole mobility, is costly and also not very eco-friendly.^17,21,26–29^ For example, the stability test (33 days at a relative humidity of 35%) shows that the Cu_2_O-HTL-based DP devices maintained more than 96% of the initial PCE than the Spiro-OMeTAD-HTL devices (84%).^30^ The simulation input parameters for the FTO, ETL, absorber, and HTL are shown in Table S1 (ESI†). They were collected from previously published experimental and computational reports, as indicated in all cases in the table. All the simulations were performed under AM 1.5G solar spectrum illumination at 300 K, while the electron and hole thermal velocities were fixed to 1 × 10^7^ cm s^−1^. The work function of FTO and Au were fixed at 4.4 eV and 5.1 eV, respectively. The band alignments at the ETL/absorber and absorber/HTL interfaces are shown in Fig. 1. The systematic device analysis was initially done without considering series and shunt resistances (parasitic resistances, *R*_series_ and *R*_shunt_).

## 3. Results and discussion

**Fig. 2:**
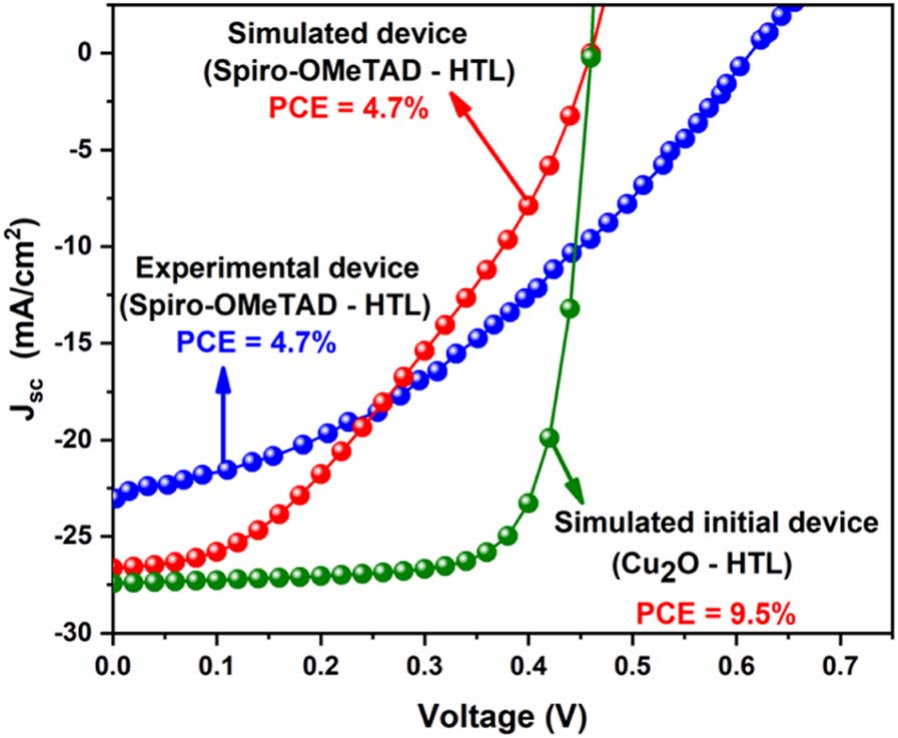
*J*–*V* characteristics of the simulated initial device using Cu_2_O-HTL as compared with the experimental device^19^ based on the conventional Spiro-OMeTAD-HTL.

Fig. 2 represents the current density–voltage characteristics (*J*–*V*) of the simulated initial device using Cu_2_O-HTL as compared with the published experimental^19^ and simulated device based on the conventional Spiro-OMeTAD-HTL under the same conditions. The simulated device with Cu_2_O-HTL showed a PCE of 9.5%, which is 2-fold higher than the experimental and simulated device with conventional Spiro-OMeTAD-HTL, revealing the strong potential of Cu_2_O and the great future of lead-free FA_4_GeSbCl_12_ DP solar cells.^19^

It is well-known that the ETL and HTL play crucial roles in the perovskite solar cells by efficiently transporting the photo-generated charge carriers from the absorber to their corresponding contacts, but also by blocking the electrons and holes towards selective charge carrier collection at their respective electrodes, preventing charge recombination at ETL/absorber and absorber/HTL interfaces. When the sunlight is illuminated on the solar cell, electrons and holes are generated in the perovskite absorber, which will be separated and subsequently collected at their respective contacts. The charge separation is mainly influenced by the conduction and valence band offsets (CBO and VBO) at the ETL/absorber and absorber/HTL interfaces, which directly govern device performance.

**Fig. 3:**
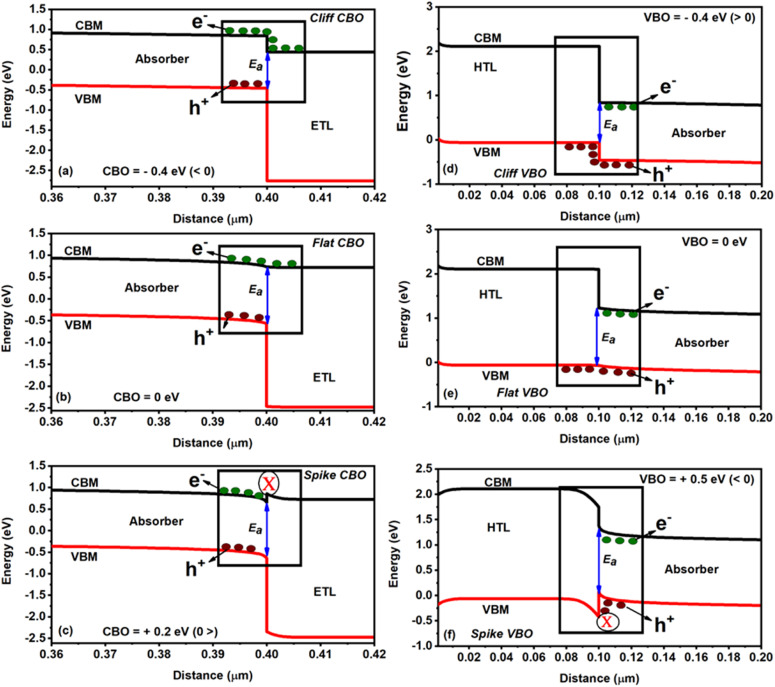
Schematic representation of the ETL/absorber and HTL/absorber interfaces with different types of barriers: (a and d) cliff-like, (b and e) nearly flat, and (c and f) spike-flat.

The CBO at the ETL/absorber interface is defined as^31^1
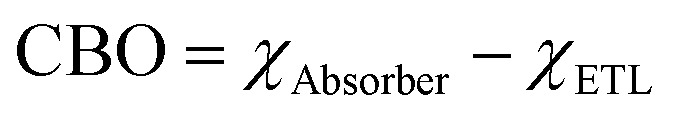
where 
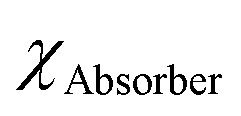
 and 
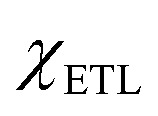
 are the electron affinities of the absorber and ETL, respectively. Three types of barriers,^32^ such as cliff-like, nearly flat, and spike-like, are observed at the ETL/absorber interface, as seen in Fig. 3a–c. A cliff-like barrier (*i.e.*, CBO is negative) is observed in Fig. 3a, occurring when 
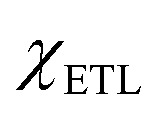
 is higher than 
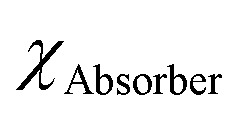
, which indicates that the conduction band minimum (CBM) of the ETL is lower than the CBM of the absorber. Fig. 3b shows a nearly flat barrier (*i.e.*, CBO is zero), meaning the zero-energy difference (*i.e.*, no barrier for the charge transfer). From Fig. 3c, a spike-like barrier (*i.e.*, CBO is positive) is observed, which arises when the CBM of the ETL is higher than the absorber (
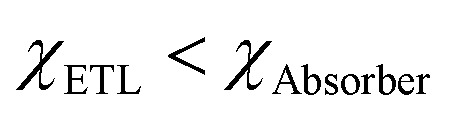
).

On the other hand, the VBO at the absorber/HTL interface is defined as^31^2
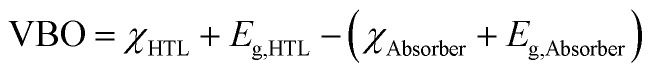
where 
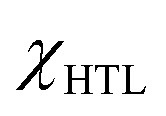
 is the electron affinity of the HTL, and *E*_g,Absorber_ and *E*_g,HTL_ are the bandgaps of the absorber and HTL, respectively. The interface of the HTL/absorber (Fig. 3d–f) showed similar barrier types as discussed for the ETL/absorber interface. Briefly, from Fig. 3d, a cliff-like barrier (VBO is negative) is observed when the valence band maximum (VBM) of the HTL is higher than the VBM of an absorber, whereas a nearly flat barrier (VBO is zero) means no band offset is seen from Fig. 3e. A spike-like barrier (VBO is positive) is detected in Fig. 3f, which is due to the VBM of HTL being lower than the absorber.

### 3.1 The impact of CBO at the ETL/absorber interface

So far, several approaches have been used to tune the properties of the ETL in the literature. For instance, doping TiO_2_ and ZnO with different elements altered their 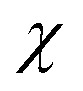, significantly changing CBO at ETL/absorber interface.^33,34^ Utilizing different ETL modifies the CBO substantially, which depends on the absorber. For example, T. Yokoyama *et al.* demonstrated the relationship between the *V*_OC_ and CBO at the ETL/absorber interface by comparing the properties of three ETLs, including Nb_2_O_5_, TiO_2,_ and SnO_2_, with two absorbers (*i.e.*, FASnI_3_ and MAFAPb(IBr)_3_).^35^ These studies provide strong evidence for the necessity of investigating the impact of CBO at the ETL/absorber interface. Therefore, we varied the 
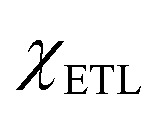
 from 3.9 to 3.3 eV with constant 
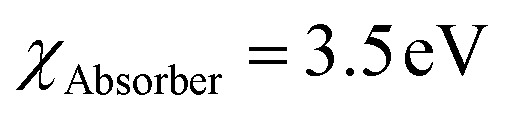
, *E*_g,Absorber_ = 1.2 eV, and *E*_g,ETL_ = 3.2 eV; as a result, CBO was modified from −0.4 to +0.2 eV.

**Fig. 4:**
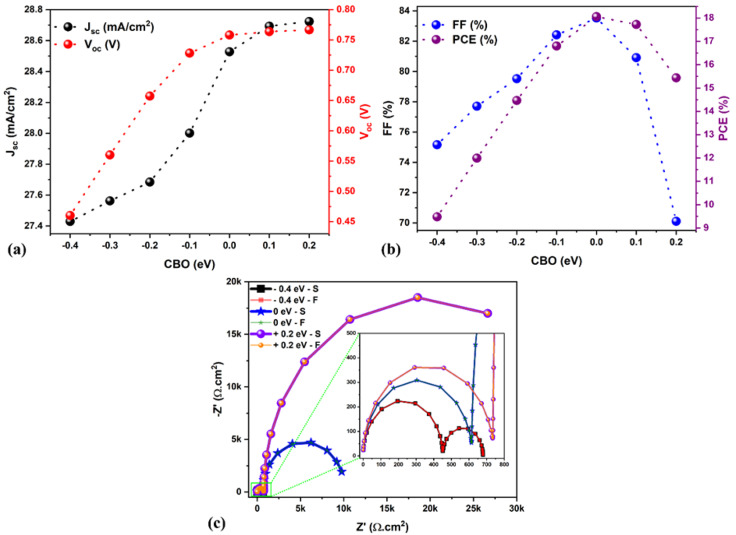
(a and b) Variations in the solar cell parameters concerning the CBO. (c) Nyquist plots for different CBO (−0.4 to 0.2 eV). Note: S denotes SCAPS impedance data, and F signifies fitted data.

Changes in the solar cell parameters (PCE, *J*_sc_, *V*_oc_, FF) as a function of CBO at the ETL/absorber interface are shown in Fig. 4a and b. The *J*_sc_ and *V*_oc_ are significantly enhanced by varying the CBO from −0.4 to +0.2 eV. In the case of negative CBO (−0.1 to −0.4 eV), CBM_ETL_ < CBM_Absorber_, creating a cliff-like barrier, which does not affect the charge carrier transport as discussed in Fig. 3a. Activation energy (*E*_a_) plays a dominant role in the interface recombination as it correlates with *V*_oc_.^32^ For example, *V*_oc_ significantly decreased when the CBO was between 0 and −0.4 eV, resulting in a reduction in the PCE from 18 to 9.5% as seen in Fig. 4a and b, which is due to a lower *E*_a_ (∼0.90 eV) as compared to *E*_g,Absorber_ as can be seen in Fig. 3a. Noticeably the FF was drastically decreased from 83 to 70% when the CBO increased beyond 0 eV as seen in Fig. 4b, which is associated with the formation of a spike-like barrier at the ETL/absorber interface (Fig. 3c), resulting in a huge reduction in the FF as there is a barrier for charge carrier transportation.^32,36^ As a consequence, the PCE was diminished from 18 to 15%, as shown in Fig. 4a and b. The *E*_a_ (see Fig. 3c) is closer or higher than *E*_g,Absorber_ in the case of the spike-like barrier. To sum up, Fig. 4a and b strongly reveal that the optimum CBO lies between 0 and 0.1 eV, which offers a PCE of ∼18%.

Impedance spectroscopy (IS) is a powerful technique that is largely used in solar cell fields.^37^ Here, Nyquist plots are simulated using SCAPS-1D to study the effect of CBO at the ETL/absorber interface, as shown in Fig. 4c. Two different relaxation regimes (*i.e.*, two different responses to frequency-modulated stimuli) with two semicircles and the horizontal axis representing real Z′ and the vertical axis denoting imaginary −Z′ were observed, similar to the other reports.^38,39^ According to the literature, the first semicircle (*i.e.*, close to the origin) is related to the high frequencies, which is attributed to the charge transport resistance (ETL/absorber and absorber/HTL interfaces). The second is associated with the lower frequencies, mainly attributed to the charge recombination within the absorber.^38–40^ In experimental devices, it is sometimes impacted by ionic transport or charge migration in the absorber. However, we firmly believe that there is no low-frequency contribution from SCAPS-1D simulation due to ionic transport. In our case, for CBO from −0.1 to −0.4 eV (negative CBO), the cliff-like barrier occurs; therefore, the lower frequency regime dominates (*i.e.*, higher recombination in the bulk absorber) compared to the other case.

The interface defects are possibly reduced in the flat and spike-like barriers than in the cliff-like, resulting in better solar cell performance. In addition to simulating impedance data using SCAPS-1D, we wanted to confront our findings to accurate data fitting using equivalent electrical circuit elements (using the reference Zview software), which allows us to extract relevant parameters such as charge transport resistance (*R*_HF_) and recombination resistance (*R*_LF_). In general, *R*_LF_ is the most important as it dominates the physical behavior of devices. Therefore, we selected three solar cells, namely CBO −0.4 eV, 0 eV, and +0.2 eV, for the fitting; the corresponding equivalent circuit diagram is shown in Fig. S1a (ESI†), and the extracted fitting plots and data are displayed in Fig. 4c and Table S2 (ESI†), respectively. The *R*_HF_ and *R*_LF_ values are significantly enhanced for the solar cell with CBO of +0.2 eV (spike-like barrier) as compared to the cliff-like barrier (−0.4 eV, see Table S2†), associated with the reduced carrier recombination. The recombination lifetime (*τ*_LF_ = *R*_LF_ × *C*_LF_) is an essential parameter for describing the recombination phenomena inside the selected solar cells. The behavior of *R*_LF_ mainly dominates *τ*_LF_ because, in the simulation, *C*_LF_ is only an approximation due to the non-consideration of ionic migration (hysteresis effect observed in the current–voltage characteristics of the solar cells). Therefore, the recombination lifetime (*τ*_LF_) has been calculated from the fitted results to gain further insight, and the results are demonstrated in Table S2.† Noticeably, a solar cell with CBO of +0.2 eV showed a longer recombination lifetime (See Table S2†), indicating slower charge carrier recombination than the other two cases (*i.e.*, 0 eV and −0.4 eV). Also, it is associated with enhanced charge carrier transport between the absorber and the ETL, and therefore, a further insightful experimental study is necessary to understand the depth mechanism.

### 3.2 The impact of VBO at the absorber/HTL interface

**Fig. 5:**
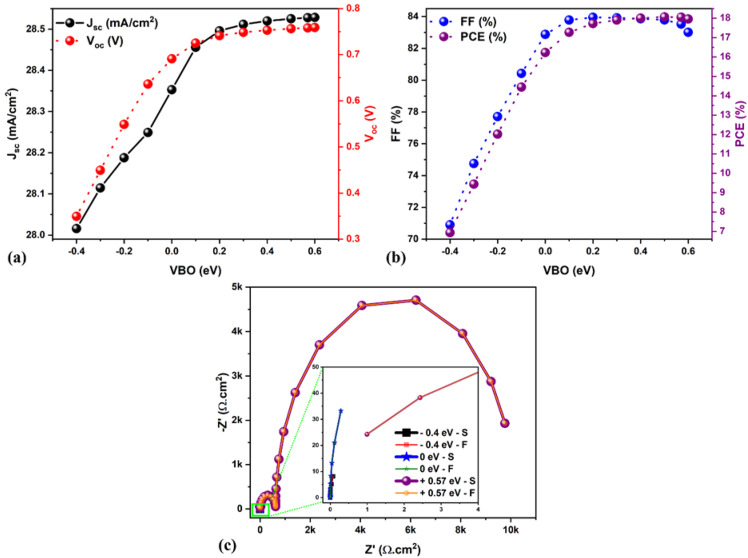
(a and b) Changes in the solar cell parameters as a function of VBO. (c) Nyquist plots for different VBO from −0.4 to +0.57 eV. Note: S signifies the SCAPS impedance data, and F denotes fitted data.

Like CBO, the VBO at the absorber/HTL interface also plays an essential and deciding role in the performance of perovskite solar cells. Therefore, in this section, the impact of VBO at the absorber/HTL interface is systematically investigated by varying the 
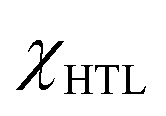
 from 2.23 to 3.23 eV without changing the *E*_g,HTL_ = 2.17 eV and 
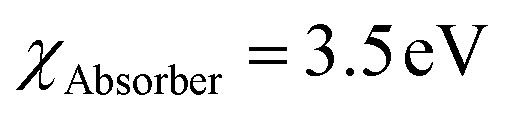
. Fig. 5a and b displays the changes in the solar cell parameters as a function of the VBO at the absorber/HTL interface. Tuning the VBO in the simulation relates to the modification of a specific property of one layer, which could be achieved in real life through the doping of the HTL material for example (such as Spiro-OMeTAD or Cu_2_O), or by surface modification using SAM layers or other types of interfacial modifiers.^41–46^ When the VBO is negative, from −0.1 to −0.4 eV (*i.e.*, VBM_HTL_ > VBM_Absorber_), a cliff-like barrier is formed at the absorber/HTL interface (see Fig. 3d). The *V*_oc_ is reduced from 0.69 to 0.34 V (see Fig. 5a), similar to the CBO negative case as discussed in the previous section. The cliff condition does not delay the photo-generated holes; however, it strongly influences the *V*_oc_ and reduces the PCE from 16 to 7% (see Fig. 5b).

On the other hand, for the positive VBO from 0 to +0.6 eV (VBM_HTL_ < VBM_Absorber_), the spike-like barrier is formed at the absorber/HTL interface, as shown in Fig. 3f. It was observed that when the VBO is positive and up to +0.57 eV, the *J*–*V* shows an increment in the *J*_sc_. However, a further increase in VBO (*i.e.*, + 0.6 eV) diminishes the FF (see Fig. 5a and b). This is possibly due to the higher VBO, which acts as a barrier for the charge carrier (*i.e.*, holes) diffusion from the HTL to the absorber side, resulting in the incomplete depletion of the absorber and, thus, the poor FF.^32^ Therefore, the optimized VBO for lead-free FA_4_GeSbCl_12_ DP solar cells lies in the range from +0.55 to +0.6 eV. Three solar cells (−0.4 eV, 0 eV, and +0.57 eV) have been selected for the IS fitting, and Fig. 5c demonstrates the Nyquist plot results, and the equivalent circuit model is shown in Fig. S1a and b.† The semicircle is highly enhanced when the VBO increases from −0.4 to +0.57 eV. Moreover, higher recombination resistance indicates decreased charge carrier recombination in bulk. The solar cell with positive VBO (+0.57 eV) showed an enhanced recombination lifetime as compared to the other two cases (see Table S2†).

An appropriate band alignment between the ETL/absorber interface (*i.e.*, the conduction band edge/LUMO level mismatch is very small) and absorber/HTL interface (*i.e.*, the valence band edge/HOMO level mismatch is minimal) facilitates the transfer of electrons (holes) from the perovskite layer into the respected ETL (HTL) rather than the non-appropriate band alignment interface.^47^ Moreover, due to the higher valence (conduction) band energy misalignment, a larger hole (electron) accumulation within the HTL (ETL) and electron (hole) accumulation in the perovskite absorber at the perovskite/HTL interface (ETL/perovskite interface) leads to higher non-radiative recombination, which is opposite to the appropriate energy alignment case.^47^ Also, the energy band misalignment creates a barrier for carrier extraction because the photo-generated carriers (electrons or holes) lose their energy through recombination, significantly reducing the charge collection efficiency.^48^ Therefore, based on our results, it is clear that having an optimum CBO and VBO is essential to achieving high-performance lead-free FA_4_GeSbCl_12_ DP solar cells. Likewise, apart from the CBO and VBO, other parameters, such as defect density and thickness, directly influence solar cell performance, and we explore these parameters in the following sections.

### 3.3 The impact of total defect density (*N*_t_) and absorber thickness

**Fig. 6:**
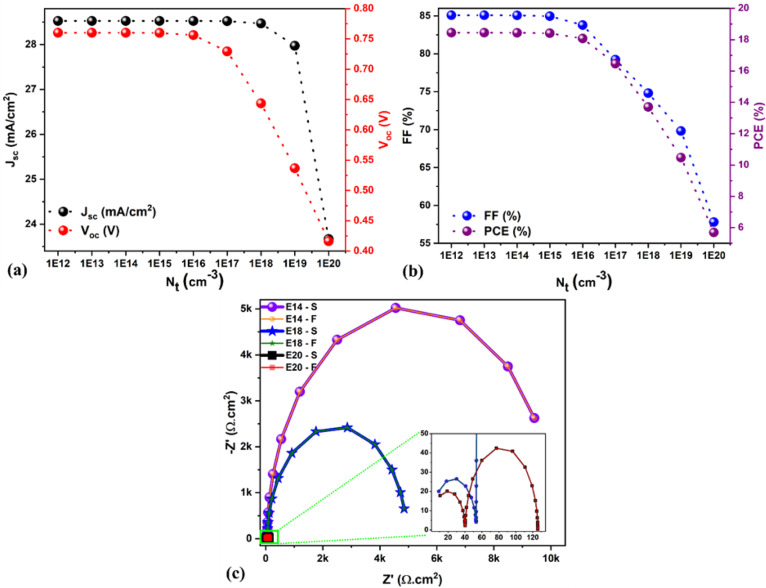
(a and b) Changes in the solar cell parameters as a function of Nt. (c) Nyquist plots for different *N*_t_. Note: S signifies the SCAPS impedance data, and F denotes fitted data.

The defect density (*N*_t_) and thickness of the absorber layer have a significant effect on the solar cell parameters due to the trapping of photogenerated charge carriers (in the former case), and considering that an increment in the absorber thickness offers more charge carrier generation due to greater photon absorption. Generally, in real solar cells, the defects are likely to be located at the surface/interface/grain boundaries, especially for Schottky, Frenkel and intrinsic point defects (such as vacancy and interstitial defects), which greatly influence the absorber electrical properties.^49–51^ Also, the fabrication environment (inert or normal atmospheric conditions) and the chosen material quality greatly help to control the defect densities to accomplish high-performance solar cells, which usually determine the recombination rates in many cases. As is well known, maintaining a very low *N*_t_ in any material is challenging. More importantly, establishing a low *N*_t_ material synthesis method is not easy. Therefore, the *N*_t_ of the absorber is varied from 1 × 10^12^ to 1 × 10^20^ cm^−3^ to understand its influence on solar cell performance. The obtained solar cell parameters are shown in Fig. 6a and b. It is evident from Fig. 6b that by decreasing the absorber *N*_t_ from 1 × 10^20^ to 1 × 10^15^ cm^−3^, there was an enhancement in the PCE from 5.7 to 18.4%. A similar trend was observed in *J*_sc_, *V*_oc_, and FF by decreasing absorber *N*_t_. This is because an increase in *N*_t_ causes a decrease in the charge carrier's diffusion length due to a reduction in the carrier lifetime, which enhances the recombination rate. No significant changes were observed in the *J*_sc_, *V*_oc_, FF, and PCE when the absorber *N*_t_ was equal to or less than 1 × 10^15^ cm^−3^ (see Fig. 6a and b). Hence, we preserved 1 × 10^14^ cm^−3^ as the optimum value as it showed a high PCE of 18.44% along with better *J*_sc_ (28.52 mA cm^−2^), *V*_oc_ (0.76 V), and FF (85%). Fig. 6c shows the Nyquist plot for three chosen solar cells, namely 1 × 10^14^ cm^−3^, 1 × 10^18^ cm^−3^, and 1 × 10^20^ cm^−3^; the associated equivalent circuit is demonstrated in Fig. S1a.† The higher *R*_LF_ (*i.e.*, lower recombination in bulk) and the enhanced recombination lifetime values were observed for a solar cell with lower *N*_t_ (1 × 10^14^ cm^−3^) as compared to the higher one (1 × 10^20^ cm^−3^, see Table S2†), which correlates well with the reduction in the PCE from 18 to ∼6% (see Fig. 6b).

**Fig. 7:**
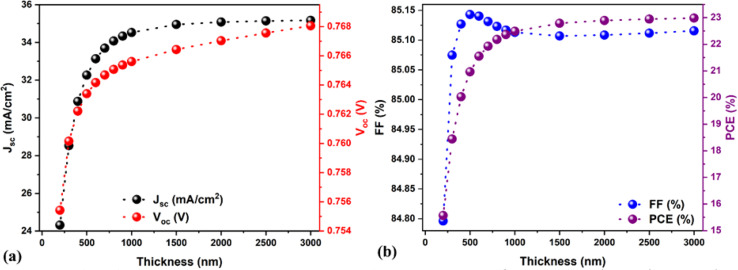
(a and b) Variations in the solar cell parameters as a function of absorber thickness.

The optimization of the absorber thickness is crucial for any solar cell. Whether in experiments or simulation, thickness mainly affects the photon collection and charge collection and directly influences solar cell parameters. Therefore, the absorber thickness was varied from 200 to 3000 nm to understand its influence on the solar cell performance. After 2000 nm, the PCE enhancement was prolonged as compared to previous ranges. Therefore, we fixed the maximal thickness at 3000 nm, and the obtained solar cell parameters are shown in Fig. 7a and b. Results show that the PCE continuously increased from 15.5 to 23% by increasing the absorber thickness from 200 to 3000 nm (see Fig. 7b). In general, the conversion of photo-generated electron–hole pairs to photo-generated current and their transport without or with reduced recombination issues happens when the absorber layer thickness is lower than the charge carrier's diffusion lengths.^52^ In this case, the solar cell demonstrated higher performance. Conversely, photon absorption is reduced if the absorber thickness exceeds the charge carrier's diffusion lengths. This is due to the photogeneration of fewer electron–hole pairs, which is detrimental to the charge carrier extraction and diminishes the solar cell performance.

According to the previously published and certified experimental reports, an absorber thickness below or equal to 1 μm is considered optimum for fabricating highly efficient PSCs.^53–55^ Therefore, we have chosen the absorber thickness of 1000 nm (PCE = 22.49%, *J*_sc_ = 34.52 mA cm^−2^, *V*_oc_ = 0.76 V, and FF = 85.1%) as an optimum value for further investigation. Fig. S2† shows the Nyquist plots for three solar cells with absorber thicknesses of 200 nm, 1000 nm, and 3000 nm, and the corresponding equivalent circuit models are shown in Fig. S1a and S1c.† Notably, the inductor (L) element (Fig. S1c†) was added to the solar cells with absorber thicknesses of 1000 and 3000 nm to get an accurate fit of SCAPS-1D data, and the extracted results are shown in Table S2.† It is worth mentioning here that a direct comparison between 200 nm and other fitted solar cells (*i.e.*, 1000 nm and 3000 nm, Fig. S2†) is not relevant because the fitted equivalent circuit model is different. According to the literature, the inductive loop in IS is related to the surface states and nonlinear accelerated kinetics of an intermediate state, which are generally associated with complex multistep dynamics.^37,56^ Therefore, finding the precise physical meaning of all the equivalent circuit model elements is necessary to elaborate the impedance spectra, which is beyond the scope of the current paper. Also, the Nyquist plots were made for the optimized solar cell with a different applied bias voltage range from 0 to 0.7 eV, as shown in Fig. S3.† The arc size drastically diminished (Fig. S3†), and the fitted results are shown in Table S2,† and the *R*_LF_ (*i.e.*, recombination resistance) values were significantly reduced (see Table S2†) by increasing the bias voltage range, which is in direct correlation with the enhanced recombination rate, as observed by other researchers.^49^

**Fig. 8:**
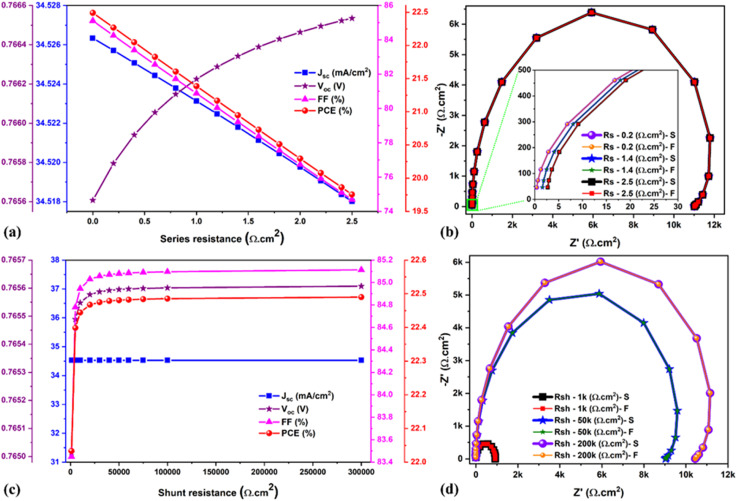
(a and c) Changes in the solar cell parameters as a function of series and shunt resistance, respectively. (b) and (d) Nyquist plots for different series and shunt resistances, respectively. Note: S signifies the SCAPS impedance data, and F denotes fitted data.

It is well-known that high-efficiency solar cells must have a low *R*_series_ and a higher *R*_shunt_. Therefore, it is clear that these parasitic resistances play a significant role and greatly impact solar cell performance. In general, the *R*_series_ increases due to the electrical resistance associated with the front and back contacts (FTO and Au), but also due to the electrical dissipation in the resistive charge transport layers (ETL and HTL) and charge-generating absorber. Meanwhile, *R*_shunt_ is affected by the different charge recombination paths, which generally occur due to defects in the layers and/or at the interfaces but also due to morphological defects (such as pinholes or voids) that generate current leakages. Therefore, the impact of parasitic resistances was systematically investigated by varying *R*_series_ from 0 to 2.5 Ω cm^2^ and *R*_shunt_ from 0 to 300 K Ω cm^2^. The chosen *R*_series_ range seems small. However, it proves how even the small *R*_series_ significantly influences solar cell performance in the relatively simplified framework of our simulations. Fig. 8a and c show the corresponding evolution of the solar cell parameters for parasitic changes in the devices and the Nyquist plots for the three chosen values, specifically 0.2 Ω cm^2^, 1.4 Ω cm^2^ and 2.5 Ω cm^2^. The efficiency of the solar cell was consistently reduced while increasing the *R*_series_ (ideal device by means *R*_series_ = 0 Ω cm^2^, PCE = 22.49% and non-ideal or realistic device having *R*_series_ = 2.5 Ω cm^2^, PCE = 19.75%), wherein *V*_oc_ remains unchanged (see Fig. 8a). The fitted results are shown in Table S2,† which exhibits the similarity between the chosen *R*_series_ from SCAPS-1D (0.2 Ω cm^2^) and the extracted *R*_series_ by IS (0.241 Ω cm^2^). Moreover, the reduction in the recombination resistance was observed even in the small series resistance range from 0.2 to 2.5 Ω cm^2^ (see Table S2†). On the other hand, the PCE was significantly enhanced while increasing the *R*_shunt_ (realistic device *R*_shunt_ = 1k, PCE = 22.0%) and ideal device (*R*_shunt_ < 200k, PCE = 22.49%) (see Fig. 8c). Fig. 8d shows the Nyquist plots for different *R*_shunt_ resistances, *i.e.*, 1k Ω cm^2^, 50k Ω cm^2^, and 200k Ω cm^2^, and the corresponding fitted results are displayed in Table S2.† While increasing the *R*_shunt_, significant enhancement was observed in the recombination resistance and lifetime values, reducing the charge carrier recombination in the absorber bulk and its interfaces. Therefore, it is clear that there are more possibilities for recombination issues with high *R*_series_ and low *R*_shunt_, resulting in detrimental device performance.

### 3.4 Tandem model

**Fig. 9:**
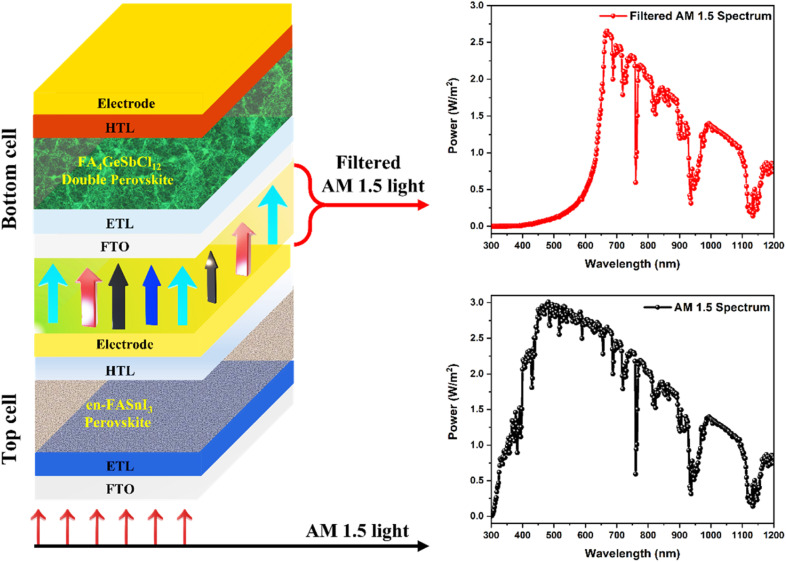
Schematic representation of the lead-free perovskite–perovskite tandem solar cell with the standard AM 1.5 spectrum for the top cell and transmitted/filtered spectrum for the bottom cell.

This section uses the simulated structure (FTO/TiO_2_/FA_4_GeSbCl_12_/Cu_2_O/Au) discussed in Fig. 1 as a bottom or narrow bandgap cell (NBGC) in the following perovskite–perovskite tandem model configuration shown in Fig. 9. Two terminal tandem (2T) or multi-junction models usually consist of two different single junction cells (*i.e.*, one is on the top and the other is on the bottom) electrically connected by an interconnecting layer. Therefore, the current through the sub-cells would be identical to achieve a good tandem performance. The top sub-cell always consists of large bandgap materials (*i.e.*, 1.7 to 2.0 eV) to absorb the higher energy photons from the solar spectrum, which transmits the lower energy photons. Therefore, narrow bandgap absorbers (*i.e.*, 1.1 to 1.6 eV) are always used in the bottom-sub-cells to collect the transmitted or filtered lower energy photons from the top-sub-cells.^57–63^

For the top sub-cell or wide bandgap cell (WBGC), an ethylenediammonium (en)-incorporated formamidinium tin iodide (simply en-FASnI_3_) perovskite absorber was adopted. The WBGC en-FASnI_3_ absorber was selected because of its lead-free composition and wide bandgap of 1.9 eV (doped with 25% of en). In addition, the en-doping increased the air stability and photoelectric properties, as proven by experimental studies.^64^ To our knowledge, the simulation of the abovementioned perovskite composition-based drift-diffusion solar cells in single or multi-junctions has not yet been published. Besides, we intended to use the formamidinium cation-based perovskite absorbers in both the top and bottom sub-cells. Therefore, WBGC with a structure of FTO/TiO_2_/en-FASnI_3_/PTAA/Au was used, and the corresponding physical input parameters were chosen from previously published experimental and theoretical reports, as displayed in Table S3.† First, the top cell was simulated by adopting the AM 1.5 spectrum with a conventional temperature of 300 K, known as a standalone condition, using drift-diffusion SCAPS-1D software.^60^ The simulated solar cell showed an excellent PCE of 9.05% in combination with *J*_sc_ = 11.42 mA cm^−2^, *V*_oc_ = 1.16 V, and FF = 68.2% as compared to the experimental results (PCE = 2.34%, *J*_sc_ = 7.64 mA cm^−2^, *V*_oc_ = 0.55 V and FF = 55.8%). However, W. Ke *et al.*^64^ demonstrated the higher performance of single-junction solar cells (PCE = 7.14%, *J*_sc_ = 22.54 mA cm^−2^, *V*_oc_ = 0.48 V and FF = 65.9%) with 10% en-doping (1.5 eV) as compared to 25%.

**Fig. 10:**
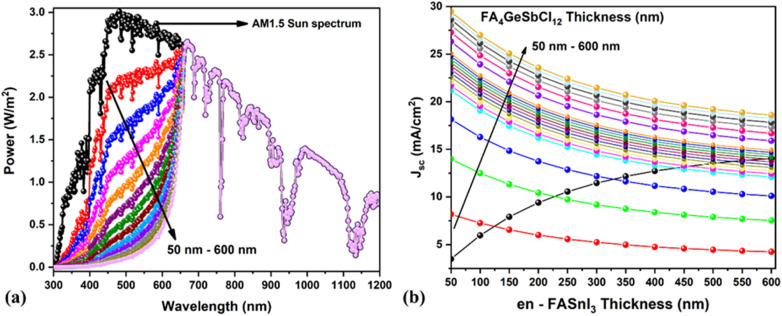
(a) Transmitted spectrum by the top WBG subcell at different thicknesses (50 to 600 nm). (b) *J*_sc_ curves of top and bottom subcells at various WBG (en-FASnl_3_) and NBG (FA_4_GeSbCl_12_) perovskite absorber thicknesses.

In this study, we have chosen 25% en-doping because it has a large bandgap (1.9 eV) and collects high-energy photons, which is more suitable for top cells in the tandem model. The current-matching condition (*i.e.*, the same current in both top and bottom cells)^65^ is the crucial factor in designing an efficient tandem model, which is usually attained *via* a tunnel recombination junction (TRJ). The ideal TRJ (which means no optoelectrical losses) between the top and bottom cells (*i.e.*, WBGC and NBGC) helps to design the tandem configuration (see Fig. 9), which is similar to our previous work.^60^ This methodology permits the simultaneous simulation of both top and bottom cells using different illumination spectra. For instance, the AM 1.5 spectrum was applied in the top WBGC. The filtered AM 1.5 spectrum was used to investigate the bottom NBGC performance (see Fig. 9). Similar to our previous publication,^60^ the transmitted AM 1.5 spectrum by the top WBGC was calculated by employing the absorption coefficient and thickness of all layers present in the top cell (shown in Fig. 10a).

Fig. 10b displays the variation in the *J*–*V* characteristics of a standalone top cell concerning perovskite layer thickness (50 to 600 nm) using the AM 1.5 spectrum. The *J*_sc_ and PCE significantly increased from 3.8 to 13.9 mA cm^−2^ and 2.8 to 11.0%, respectively. The systematic study was conducted by varying the absorber thickness of the top WBGC and bottom NBGC to find the best current-matching condition. Fig. 10b demonstrates the reduction in the *J*_sc_ of the bottom NBGC due to the small number of photons striking the NBGC as the top cell absorber thickness increases. For example, the higher photo-current passed from bottom subcells when the top layer thickness was 50 nm, and *vice versa* (photo-current diminished at a higher thickness of 600 nm). Hence, the thickness optimization helped to achieve the same *J*_sc_ values in both subcells (*i.e.*, top and bottom cells), which is crucial in obtaining a potential tandem cell. For instance, the same *J*_sc_ of 13.99 mA cm^−2^ was obtained for the top and bottom cells of en-FASnI_3_ and FA_4_GeSbCl_12_ at 588.4 nm and 260 nm, respectively.

**Fig. 11:**
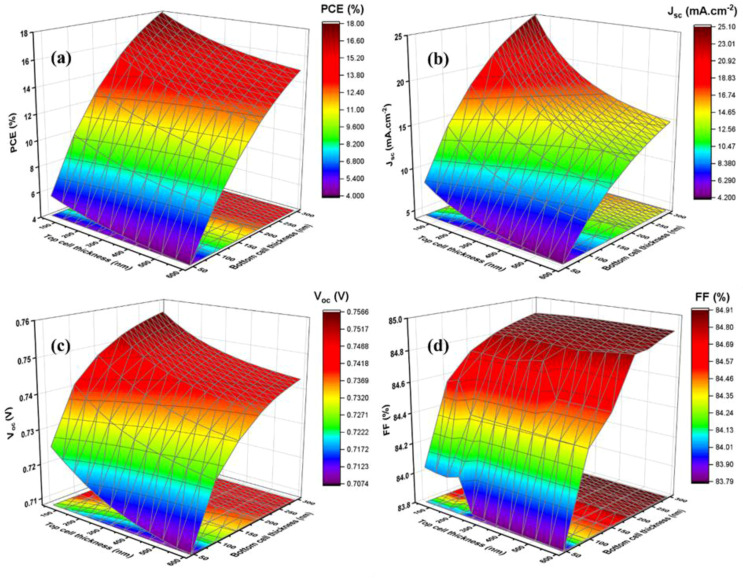
Changes in the solar cell parameters (PCE (a), *J*_sc_ (b), *V*_oc_ (c), and FF (d)) of the bottom NBG cell concerning WBGC thicknesses.

**Fig. 12:**
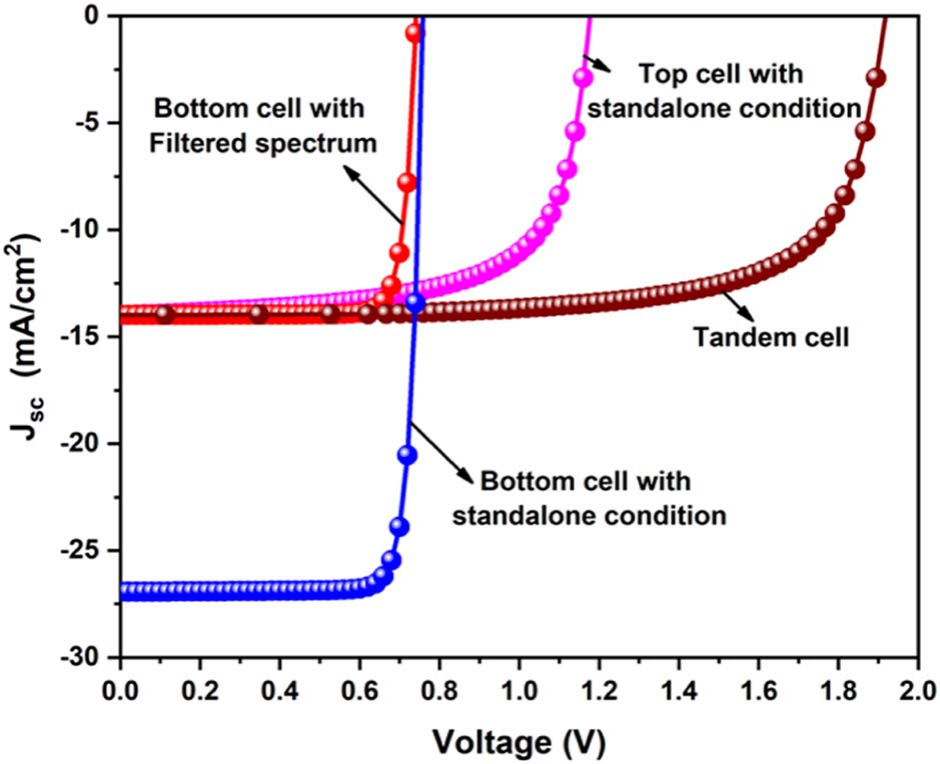
*J*–*V* curves of the top and bottom cells with the standalone condition, the bottom cell with the transmitted filtered spectrum, and the finalized tandem cell.

Fig. 11a–d demonstrates the solar cell parameters concerning the bottom NBGC absorber layer thickness using the transmitted filtered spectrum. The best PCE of 14.01% (top subcell1 – 1.1%) was obtained at 260 nm thickness of NBGC with an outstanding current matching condition for simulating the two-terminal tandem devices. The chosen thickness of the top WBGC perovskite layer reduced the incident light (*i.e.*, AM 1.5 spectrum) power from 1000 W m^−2^ to 628.4 W m^−2^ due to the transmitted filtered spectrum. After finding the suitable current-matching conditions, the *J*–*V* curve of the tandem device was calculated by adding the voltage at equal current points. Fig. 12 shows the *J*–*V* curves of the top and bottom cells with the standalone conditions, the bottom cell with the transmitted filtered spectrum, and finalized tandem cell. Briefly, the final lead-free perovskite–perovskite tandem cell (*i.e.*, FA_4_GeSbCl_12_–en-FASnI_3_) delivered a PCE as high as 19.05% with *J*_sc_ of 13.99 mA cm^−2^, *V*_oc_ of 1.92 V and FF of 71.4%.

The external quantum efficiencies (EQE) of both top (WBGC) and bottom (NBGC) subcells with the standard and filtered (transmitted) AM 1.5 spectrum are demonstrated in Fig. S4.† The top WBGC has a higher bandgap (1.9 eV) perovskite absorber that efficiently absorbs the shorter wavelength radiations up to 650 nm. Simultaneously, the longer wavelengths are filtered from the top cell and then transmitted to the NBGC as it has a lower bandgap (1.3 eV) perovskite absorber, which absorbs longer wavelength radiations >560 nm (see Fig. S4†). Table 1 compares the solar cell parameters of lead-free perovskite–perovskite tandem cells with the previously published reports from the literature.

**Table 1:** Solar cell parameters of the top WBGC, bottom NBGC, and the lead–free perovskite - perovskite tandem cell compared with lead–free tandem works published so far in the literature

Device	*J*_sc_ (mA cm^−2^)	*V*_oc_ (V)	FF (%)	PCE (%)	Ref.
Top cell (under AM 1.5 spectrum)	13.99	1.18	67.28	11.1	This work
Bottom cell (under transmitted filtered AM 1.5 spectrum)	13.99	0.75	84.87	14.0	
Tandem cell (current matching condition)	13.99	1.92	71.44	19.0	
MAGeI_3_–FAMASnGeI_3_ cell	28.36	1.07	84.46	26.7	66
MAGeI_3_–FASnI_3_ cell	14.70	2.63	79.80	30.8	58
MASnIBr_2_–MASnI_3_ cell	13.94	1.89	60.57	15.6	59
MAGeI_3_–MASnI_3_ cell	10.40	2.10	71.43	19.1	67

Recently, Arman U. Duha *et al.* achieved a PCE of 30.8% by adopting MAGeI_3_ (1 μm top cell) with FASnI_3_ (1.6 μm bottom cell).^58^ However, as discussed before, in realistic tandem models, the thicker top cell reduces the striking photons to the NBG bottom subcell. Neelima Singh *et al.* simulated the MAGeI_3_ (200 nm) – FAMASnGeI_3_ (300 nm) lead-free perovskite–perovskite tandem model with a PCE of 26.7%.^66^ However, the authors did not implement a filtered spectrum strategy. They used the standard AM 1.5 spectrum to optimize the cells, which could be a reason for the higher *J*_sc_ (28.36 mA cm^−2^) and superior performance. Furthermore, Saugata Sarker *et al.* (MAGeI_3_ (255 nm top cell) – MASnI_3_ (300 nm bottom cell))^67^ and S. Abdelaziz *et al.* (MASnIBr_2_ (320 nm top cell) – MASnI_3_ (350 nm bottom cell))^59^ adopted a filtered spectrum approach to achieve a current matching condition for their lead-free tandem models and accomplished a PCE of 19.1%^67^ and 15.6%,^9^ respectively, similar to our tandem solar cell performance.

The bandgap of the chosen perovskite absorber, ETL, and HTL mitigate losses due to carrier thermalization in the realistic tandem cells.^68^ Moreover, controlling the parasitic absorption and reflections from additional transparent reflections and interfaces is essential for better tandem cell performance. Importantly, minimizing the halide segregation and the recombination issues will prevent voltage loss, especially in the WBG sub-cell side.^69^ Adopting a suitable interconnecting layer in the tandem cell and the energy band alignment between the layers enhances the PCE.^70^ Overall, perovskite absorbers, ETL, HTL, interconnecting layer, fabrication methods, and conditions are crucial for avoiding optical and electrical losses and recombination issues at the interface/surface, which could unquestionably improve the tandem cell performance.

## 4. Conclusion

In this work, we studied the performance of a new, lead-free, formamidinium germanium-antimony halide (FA_4_GeSbCl_12_) DP solar cell using a drift-diffusion SCAPS-1D simulation. We systematically tuned the CBO (−0.4 to +0.2 eV) and VBO (−0.4 to +0.57 eV) to find the suitable band alignment between ETL/absorber and HTL/absorber interfaces. The impacts of defect density (1 × 10^14^–1 × 10^20^ cm^−3^) and the absorber thickness (200–3000 nm) on the solar cell performance have been investigated in detail. The findings are summarized as follows:

• The optimization of the CBO and VBO between ETL/absorber and HTL/absorber interfaces shows that the enhanced PCE of over 18% (*J*_sc_ = 28.5 mA cm^−2^, *V*_oc_ = 0.75 V, and FF = 83.78%) for the DP solar cell is highly attributed to the CBO and VBO ranging from 0 to 0.1 eV and 0.5 to 0.6 eV, respectively, which facilitates the electron and hole extraction from the absorber and reduces the recombination at the interfaces.

• The optimized absorber defect density and thickness were found at 1 × 10^14^ cm^−3^ and 1000 nm, demonstrating an improved efficiency of 18.45% (*J*_sc_ = 28.5 mA cm^−2^, *V*_oc_ = 0.76 V, and FF = 85.07%) and 22.5% (*J*_sc_ = 34.52 mA cm^−2^, *V*_oc_ = 0.76 V, and FF = 85.1%).

• The fitted SCAPS-1D impedance data with the Zview software helped find the appropriate equivalent circuit model to extract IS parameters, allowing us to understand the physical mechanism of solar cells. Besides, the fitted results firmly revealed that the higher PCE solar cell showed an enhanced recombination resistance and longer recombination lifetime.

• The solar cell efficiency was consistently reduced while increasing the *R*_series_; for example, *R*_s_ = 0 Ω cm^2^ (PCE = 22.5%) and *R*_s_ = 2.5 Ω cm^2^ (PCE = 19.75%) prove how even the small *R* series significantly influences solar cell performance.

• Finally, the tandem model was constructed using the en-FASnl_3_ perovskite top cell (588.4 nm) with the FA_4_GeSbCl_12_ bottom cell (260 nm) using a filtered spectrum strategy with an excellent PCE of 19% with photovoltaic parameters of *J*_sc_ = 13.99 mA cm^−2^, *V*_oc_ = 1.92 V, and FF = 71.44%.

Overall, this simulated work provides a roadmap for developing low-cost, solution-processed, non-toxic, single, and multi-junction perovskite solar cells with improved PCE. Further insightful experimental studies are necessary to determine the issues at the interfaces/surfaces, which will help to enhance the device's performance beyond 20%.

## Conflicts of interest

There are no conflicts to declare.

## Supplementary Material

RA-013-D3RA03102K-s001
