# Supplementary material for: Lead-free, formamidinium germanium-antimony halide (FA4GeSbCl12) double perovskite solar cells: the effects of band offsets
Source: RSC Adv. 2023 Aug 25;13(36):25483–96. doi: 10.1039/d3ra03102k (PMC10450393; doi:10.1039/d3ra03102k)
Supplement: RA-013-D3RA03102K-s001 [file RA-013-D3RA03102K-s001.pdf]

Electronic Supplementary Material (ESI) for RSC Advances.
This journal is © The Royal Society of Chemistry 2023

# Supporting Information

## Lead-free, formamidinium germanium-antimony halide ($FA_4GeSbCl_{12}$) double perovskite solar cells: effect of band offsets

Karthick Sekar,[a,b,*] Latha Marasamy,[c] Sasikumar Mayarambakam,[d,*] Hesham Hawashin,[a] Mohamad Nour,[b] Johann Bouclé[a]

(a) Univ. Limoges, CNRS, XLIM, UMR 7252, F-87000, Limoges, France.
(b) GREMAN UMR 7347, Université de Tours, CNRS, INSA Centre Val de Loire, 37071 Tours, France.
(c) Facultad de Química, Materiales-Energía, Universidad Autónoma de Querétaro, Santiago de Querétaro, Querétaro, C.P. 76010, Mexico.
(d) Department of Chemistry, Indian Institute of Science Education and Research (IISER)-Tirupati, Tirupati, 517507, A.P., India.

**Table S1**. SCAPS-1D input parameters of FTO, $TiO_2$, $FA_4GeSbCl_{12}$ and $Cu_2O$.

| **Parameters** | **FTO (TCO)** | **$TiO_2$ (ETL)** | **$FA_4GeSbCl_{12}$ (Absorber)** | **$Cu_2O$ (HTL)** |
|---|---|---|---|---|
| **Thickness (nm)** | 500 | 30 | 300 | 100 |
| **Bandgap, $E_g$ (eV)** | 3.50 | 3.20 | 1.3 | 2.17 |
| **Affinity, $\chi$ (eV)** | 4.00 | 3.9 | 3.50 | 3.2 |
| **Permittivity, $\varepsilon_r$** | 9.00 | 9.00 | 2.59 | 7.11 |
| **Effective density of states at CB, $N_c$ ($cm^{-3}$)** | $2.2\times10^{18}$ | $2.2\times10^{18}$ | $2.2\times10^{18}$ | $2.02\times10^{17}$ |
| **Effective density of states at VB, $N_v$ ($cm^{-3}$)** | $1.8\times10^{19}$ | $1.8\times10^{19}$ | $2.0\times10^{18}$ | $1.1\times10^{19}$ |
| **Mobility of electrons, $\mu_n$ ($cm^2V^{-1}s^{-1}$)** | 20 | 20 | 2268 | 200 |
| **Mobility of holes, $\mu_p$ ($cm^2V^{-1}s^{-1}$)** | 10 | 10 | 478 | 80 |
| **Density of n-type doping, $N_D$ ($cm^{-3}$)** | $1.0\times10^{18}$ | $1.0\times10^{19}$ | 0 | 0 |
| **Density of p-type doping, $N_A$ ($cm^{-3}$)** | 0 | 1 | $1.0\times10^{15}$ | $1.0\times10^{18}$ |
| **Density of defects, $N_t$ ($cm^{-3}$)** | $1.0\times10^{15}$ | $1.0\times10^{15}$ | $1.0\times10^{16}$ | $1.0\times10^{15}$ |
| **Reference** | [1] | [2,3] | [4,5] | [2,6] |

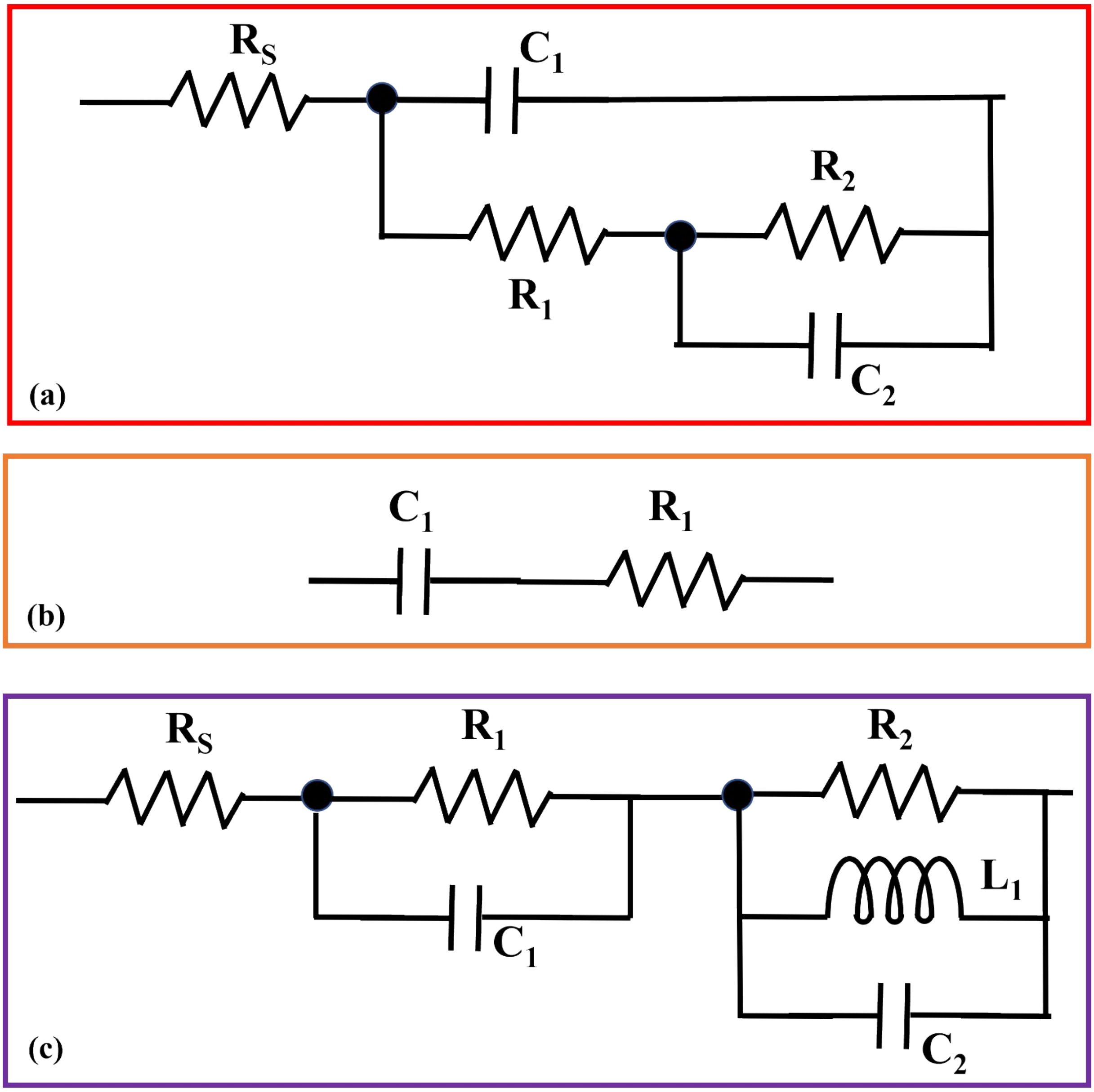


**Figure S1**. Equivalent circuit for Nyquist plots of EIS spectra with respect to the SCAPS-1D impedance data under AM 1.5 spectrum. (a) CBO (- 0.4 eV, 0 eV, + 0.2 eV), VBO (+ 0.57 eV), $N_t$ (1E14 cm$^{-3}$, 1E18 cm$^{-3}$, 1E20 cm$^{-3}$) and absorber thickness (200 nm), (b) VBO (- 0.4 eV and 0 eV), and (c) absorber thickness (1000 nm and 3000 nm), 1000 nm (0 V, 0.3 eV, 0.6 eV), Series resistance (0.2 Ω.cm$^2$, 1.4 Ω.cm$^2$, 2.5 Ω.cm$^2$) and Shunt resistance (1k Ω.cm$^2$, 50k Ω.cm$^2$, 200k Ω.cm$^2$), respectively.

**Table S2**. Fitted results from the Nyquist Plot of simulated solar cell with different selected parameters (CBO, VBO, $N_t$, absorber thickness, bias voltage, series and shunt resistances).

| | | Rs Ω.cm² | L | R Ω.cm² (HF) | CPE or C F/cm² (HF) | R Ω.cm² (LF) | CPE or C F/cm² (LF) | tau (LF) |
|---|---|---|---|---|---|---|---|---|
| CBO (eV) | - 0.4 | 0.004 | | 451 | 6.403E-9 | 228 | 1.341E-5 | 0.0031 |
| | 0 | 0.004 | | 617 | 6.570E-9 | 9552 | 3.512E-6 | 0.0336 |
| | + 0.2 | 0.004 | | 739 | 6.566E-9 | 37041 | 2.812E-6 | 0.1042 |
| VBO (eV) | - 0.4 | | | | | 0.0195 | 2.751E-5 | 5.3745E-7 |
| | 0 | | | | | 0.014 | 3.346E-5 | 4.6840E-7 |
| | +0.57 | 0.045 | | 618 | 6.562E-9 | 9554 | 3.512E-6 | 0.0336 |
| Nt (cm⁻³) | E14 | 0.066 | | 53.45 | 6.606E-9 | 10100 | 4.406E-6 | 0.0445 |
| | E18 | 0.044 | | 53.31 | 6.604E-9 | 4889 | 4.437E-6 | 0.0217 |
| | E20 | 0.044 | | 40 | 6.557E-9 | 85.71 | 8.531E-6 | 7.3115E-4 |
| Absorber Thickness (nm) | 200 | 0.051 | | 20 | 9.85E-9 | 9172 | 1.196E-5 | 0.1097 |
| | 1000 | 0.041 | 0.478 | 11006 | 4.32E-9 | 2035 | 1.718E-8 | 3.4969E-5 |
| | 3000 | 0.046 | 0.505 | 10327 | 4.39E-9 | 2131 | 1.722E-8 | 3.6706E-5 |
| 1000 (nm) | 0 V | 0.041 | 0.478 | 11006 | 4.32E-9 | 2035 | 1.718E-8 | 3.4969E-5 |
| | 0.3 V | 0.041 | 0.333 | 6738 | 5.42E-9 | 1525 | 1.872E-8 | 2.8552E-5 |
| | 0.6 V | 0.039 | 0.018 | 372 | 7.46E-9 | 99.63 | 2.948E-8 | 2.9371E-6 |
| $R_{series}$ (Ω.cm²) | 0.2 | 0.241 | 0.477 | 11006 | 4.315E-9 | 2031 | 1.726E-8 | 3.5053E-5 |
| | 1.4 | 1.442 | 0.477 | 11005 | 4.313E-9 | 2029 | 1.730E-8 | 3.5104E-5 |
| | 2.5 | 2.543 | 0.473 | 11004 | 4.273E-9 | 1996 | 1.797E-8 | 3.5878E-5 |
| $R_{shunt}$ (Ω.cm²) | 1k | 0.044 | 0.0026 | 917 | 3.498E-9 | 10.93 | 2.716E-7 | 2.9688E-6 |
| | 50k | 0.044 | 0.3000 | 9029 | 4.074E-9 | 1276 | 2.267E-8 | 2.8924E-5 |
| | 200k | 0.041 | 0.4217 | 10419 | 4.145E-9 | 1724 | 2.063E-8 | 3.5571E-5 |

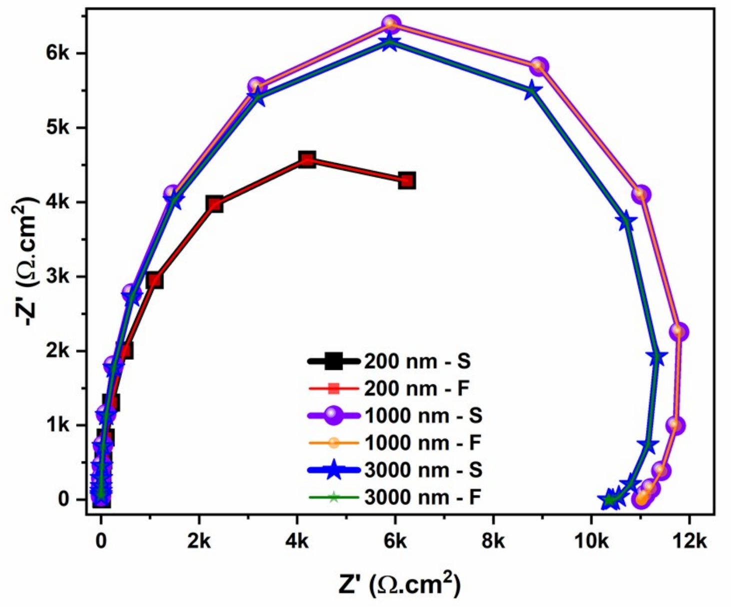


**Figure S2**. Nyquist plots as a function of absorber thicknesses. Note: S signifies the SCAPS impedance data, and F denotes fitted data.

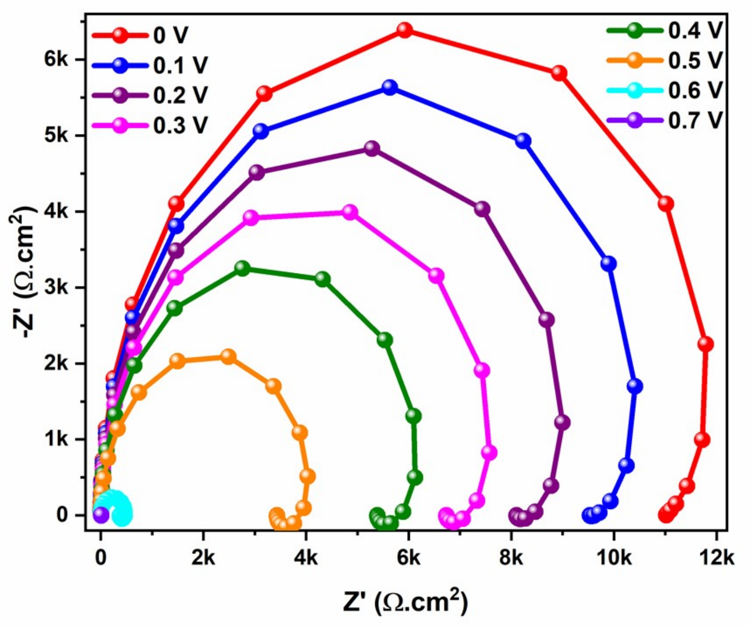


**Figure S3**. Nyquist plots of solar cell with 1000nm absorber thickness measured at different bias voltage from 0 to 0.7 V.

**Table S3**. SCAPS-1D input parameters of FTO, $TiO_2$, en-$FASnI_3$ and PTAA.

| Parameters | FTO (TCO) | $TiO_2$ (ETL) | en-$FASnI_3$ (Absorber) | PTAA (HTL) |
|---|---|---|---|---|
| **Thickness (nm)** | 500 | 30 | 300 | 100 |
| **Bandgap,** $E_g$ **(eV)** | 3.50 | 3.20 | 1.9 | 2.96 |
| **Affinity,** $\chi$ **(eV)** | 4.00 | 3.9 | 3.520 | 2.3 |
| **Permittivity,** $\varepsilon_r$ | 9.00 | 9.00 | 8.20 | 9 |
| **Effective density of states at CB,** $N_c$ ($cm^{-3}$) | $2.2\times10^{18}$ | $2.2\times10^{18}$ | $2.2\times10^{18}$ | $2.0\times10^{21}$ |
| **Effective density of states at VB,** $N_v$ ($cm^{-3}$) | $1.8\times10^{19}$ | $1.8\times10^{19}$ | $2.0\times10^{18}$ | $2.0\times10^{21}$ |
| **Mobility of electrons,** $\mu_n$ ($cm^2V^{-1}s^{-1}$) | 20 | 20 | 22 | 1 |
| **Mobility of holes,** $\mu_p$ ($cm^2V^{-1}s^{-1}$) | 10 | 10 | 22 | 40 |
| **Density of n-type doping,** $N_D$ ($cm^{-3}$) | $1.0\times10^{18}$ | $1.0\times10^{19}$ | 0 | 0 |
| **Density of p-type doping,** $N_A$ ($cm^{-3}$) | 0 | 1 | $7.0\times10^{16}$ | $1.0\times10^{18}$ |
| **Density of defects,** $N_t$ ($cm^{-3}$) | $1.0\times10^{15}$ | $1.0\times10^{15}$ | $2.0\times10^{15}$ | $1.0\times10^{15}$ |
| **Reference** | [1] | [2,3] | [7–9] | [6,9] |

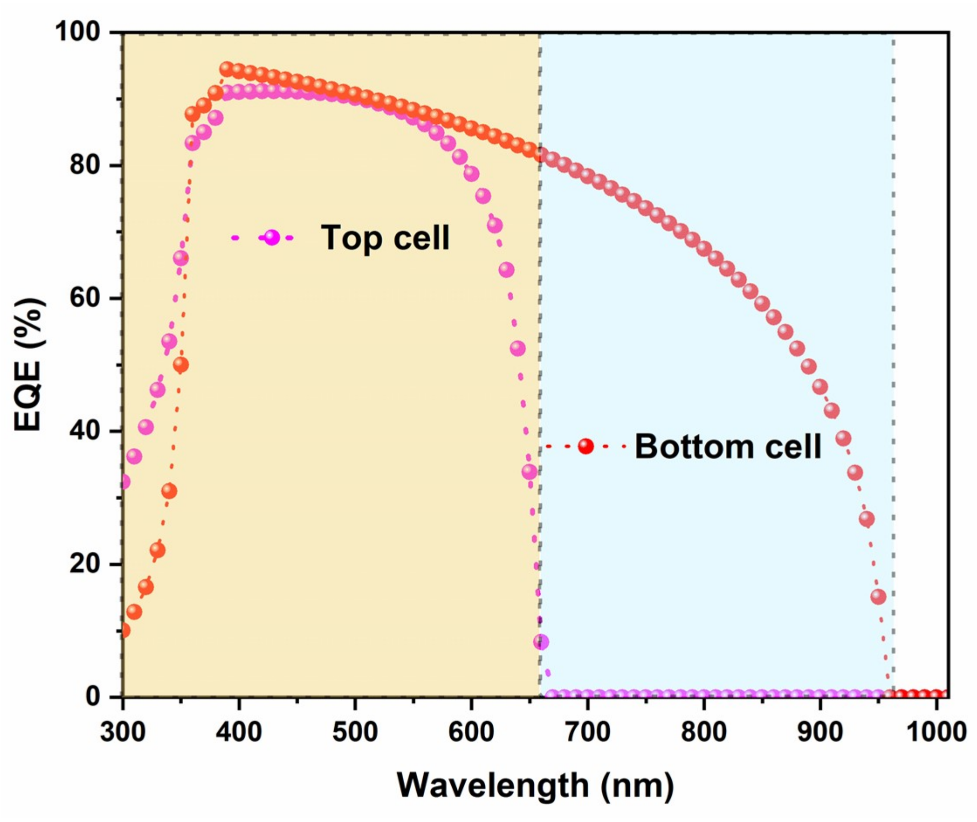


**Figure S4**. EQE of top and bottom cells.

## References

1 S. Karthick, S. Velumani and J. Bouclé, *Sol. Energy*, , DOI:10.1016/j.solener.2020.05.041.

2 A. A. Kanoun, M. B. Kanoun, A. E. Merad and S. Goumri-Said, *Sol. Energy*, 2019, **182**, 237–244.

3 A. U. Duha and M. F. Borunda, *Opt. Mater. (Amst).*, 2022, **123**, 111891.

4 C. Walkons, R. Murshed and S. Bansal, *Sol. RRL*, 2020, **4**, 2000299.

5 W. B. Dai, S. Xu, J. Zhou, J. Hu, K. Huang and M. Xu, *Sol. Energy Mater. Sol. Cells*, 2019, **192**, 140–146.

6 S. Karthick, J. Bouclé and S. Velumani, *Sol. Energy*, 2021, **218**, 157–168.

7 W. Ke, C. C. Stoumpos, M. Zhu, L. Mao, I. Spanopoulos, J. Liu, O. Y. Kontsevoi, M. Chen, D. Sarma, Y. Zhang, M. R. Wasielewski and M. G. Kanatzidis, *Sci. Adv.*, , DOI:10.1126/sciadv.1701293.

8 S. Abdelaziz, A. Zekry, A. Shaker and M. Abouelatta, *Opt. Mater. (Amst).*, , DOI:10.1016/j.optmat.2020.109738.

9 T. Yokoyama, Y. Nishitani, Y. Miyamoto, S. Kusumoto, R. Uchida, T. Matsui, K. Kawano, T. Sekiguchi and Y. Kaneko, *ACS Appl. Mater. Interfaces*, 2020, **12**, 27131–27139.
